# Supplementary material for: Evaluating Iodine-125 DNA Damage Benchmarks of Monte Carlo DNA Damage Models
Source: Cancers (Basel). 2022 Jan 18;14(3):463. doi: 10.3390/cancers14030463 (PMC8833774; doi:10.3390/cancers14030463)
Supplement: Supplementary file 1 [file cancers-14-00463-s001.zip › cancers-1535945-supplementary.pdf]

cancers

Supplementary Material

Evaluating Iodine-125 DNA Damage Predictions for DNA Physics Models

Shannon J. Thompson, Aoife Rooney, Kevin M. Prise and Stephen J. McMahon *

1. Notes on Models

As noted in the main text, three different Geant4-DNA constructors are considered, Opt2, Opt4 and Opt6. The major difference between these constructors is their electron interaction models which simulate elastic and inelastic interactions with differing models and energy limits. Opt2 simulates elastic interactions through a partial wave model and inelastic interactions through the Emfietzoglou dielectric model. Opt4 updates these interactions using the Uehara screened Rutherford and Emfietzoglou-Kyriakou models for elastic and inelastic respectively. Opt6 incorporates interactions from the CPA100 track structure code, using the independent atom method model for elastic interactions, and the relativistic binary encounter Bethe model and dielectric model for inelastic ionisation and excitation interactions respectively. Opt2 also considers inelastic sub-excitation interactions down to electron energies of 2 eV. These differences lead to significant differences in the distribution of interactions, as described in the main text, and consequently optimum physics parameters.

2. Energy Range for SSB Production

As discussed in the main text, rather than using a distinct threshold for defining an SSB, many models make use of an energy-dependent probability, which is 0 below some lower threshold, and increases linearly to a value of 1 at some upper threshold. For example, many models have used a combination of a lower threshold of 5 eV and an upper threshold of 37.5 eV.

The impact of such a threshold was also investigated in this work, by reproducing the analysis and fitting presented in the main work for such a probabilistic threshold model. Instead of being given by a single threshold energy value, E_{thresh}, two values are now required, specifying the extent of the range between damage probabilities of 0 and 1. For the purposes of this analysis, we will define these in terms of the central energy of the window, E_{centre}, and the energy width of the range, w, both of which are defined in eV. So, for example, a range beginning at 15 eV and reaching 1 at 25 eV would have a central value of $E_{thresh} =$ 20 eV, and a width of $width = 10$ eV.

Fits were performed in this work for widths ranging from 0 to 20 eV. The fit which was produced for the 20 eV width is shown in Figure (FIG)a. This has similar overall performance to that seen in the main text for a single threshold. Indeed, across all energy range widths considered there was little impact on fit quality compared to the single threshold, suggesting that appropriate selection of parameters can yield very similar results regardless of the choice of model here.

Figure (FIG)b shows how the best-fitting central value changes with increasing energy width. It can be seen that for small widths (w less than approximately 7.5 eV), there is little impact on E_{centre}, while above this value the central energy appears to increase approximately linearly with increasing w. Fitting linear regressions to the central energy above 7.5 eV window sizes suggests that all models have a slope consistent with $0.5w$.

This means that, since the lower bound of the damage window is given by $E_{centre} - 0.5w$, that as the energy width increases above a certain point, the best-fitting threshold increases so as to keep the lower bound of the energy window constant. This can be shown in Figure (FIG)c, which shows the upper and lower bounds of the best-fitting energy

windows. It can be seen that while this increases approximately symmetrically at low widths, above 7.5 eV the lower bound is approximately constant, while the upper bound increases much more rapidly. This suggests that best-fitting models are those which do not allow any breaks below some energy deposit.

In particular, it can be seen that the minimum energies (ranging from 26 to 29 eV across the different models) correspond well with a number of small peaks which can be seen in the energy deposit histogram, which relate to combinations of two of the larger ionisation potentials of water. As in the single threshold model, this suggests that the best-fitting values may be very sensitive to particular features of the way radiation interacts with water, and their exact values may need to be treated with care when extrapolating to real biological materials.

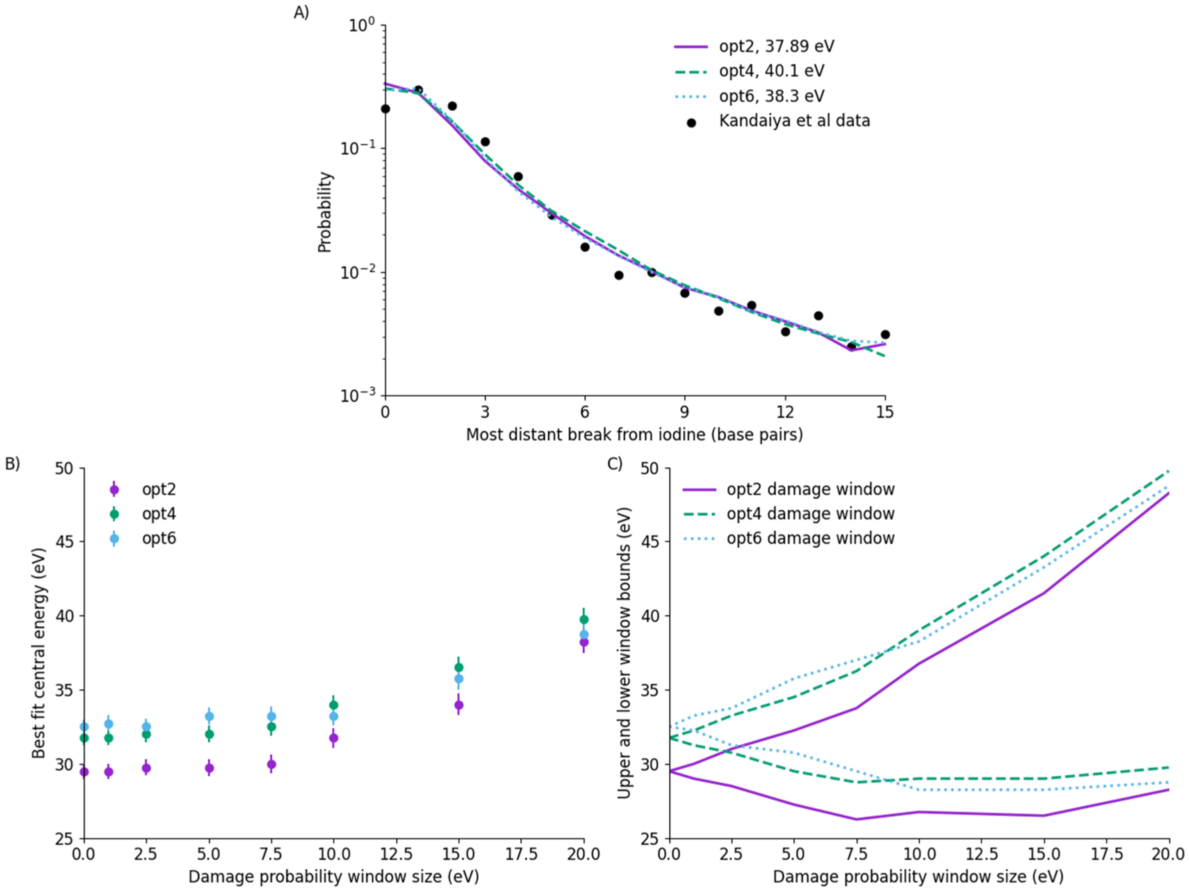

Figure S1. Physical damage fitting with different threshold ranges. (**A**) Best-fitting curves for damage simulated with a 20 eV wide threshold window. In all cases models were able to effectively reproduce data, with some increases in central value of energy threshold (legend). (**B**) Central energy value, E_{centre}, for different widths. It can be seen that these values are initially independent of w, but begin to increase as w becomes larger. (**C**) Damage window bounds for each model, with the lower curve representing the energy where SSB probability begins to increase above 0, and the upper where it becomes 1. Notably, for all models the lower threshold is roughly constant above a certain window size, while the upper bound increases rapidly.

SSB Probability Normalisation

As noted in the main text, a major challenge with interpreting the SSB distribution data in this work is the inherent normalisation in the observed data preventing the quantification of the absolute yield of breaks. Instead, measured intensities of bands corresponding to different fragment sizes are quantified and normalised to give a fragment size probability distribution.

While simulations can compare to this probability distribution, this leads to ambiguities in the interpretation of data, as discussed in the main text. One option to address this would be to attempt to convert from the fragment size distribution to an absolute break distribution, to resolve this issue. However, this is not without challenges.

In Kandaiya et al, the authors denoted the probability distribution of fragment sizes as f_i, where i was the number of nucleotides to the ^{32}P-labelled end of the DNA strand, and thus size of the fragment in base pairs. To convert this into p_i, the probability of an SSB occurring at this position, they noted that for an fragment of size i to be visible, there can be no breaks at any points closer to the 32P. Thus fragment probabilities can be expressed as:

$$f_i = p_i(1 - p_{i-1}) \dots (1 - p_0)$$

This can be rearranged to give:

$$p_i = \frac{f_i}{(1 - p_{i-1}) \dots (1 - p_0)} \quad (1)$$

In principle this can then be solved recursively, by assuming that for all undetected fragments $p_i = 0$, and thus for the first detected fragment it can be approximated as $p_1 = f_1$, and the analysis can proceed from there. This led to an estimate of significantly higher SSBs closer to the iodine, as expected, peaking with a 100% break rate at the site of the iodine.

However, the overall response of this system, particular for sites near the iodine, is under-constrained by the available data. Consequently a range of different SSB distributions are equally consistent with these fragment sizes, and the obtained values can be strongly influenced by the chosen interpretation. In this particular approach, the above assumptions lead in all cases to high SSB yields in the vicinity of the iodine, and a value of 1 at the largest nucleotide size, regardless of the underlying probability distribution.

To illustrate this, consider the estimation of the first few break probabilities, with f_0 being the smallest fragment size (most distant SSB) detected. From the above assumption, we then have:

$$p_0 = f_0$$

Then, applying the formula above for the second and third bases, we have:

$$p_1 = \frac{f_1}{(1 - p_0)} = \frac{f_1}{(1 - f_0)}$$

$$p_2 = \frac{f_2}{(1 - p_0)(1 - p_1)} = \frac{f_2}{(1 - f_0)\left(1 - \frac{f_1}{1 - f_0}\right)} = \frac{f_2}{(1 - f_0)\left(\frac{1 - f_0 - f_1}{1 - f_0}\right)} = \frac{f_2}{(1 - f_0 - f_1)}$$

It can be seen that, based on the initial assumptions, p_2 depends on f_2 divided by 1 minus the sum of the two previous fragment probabilities, f_0 and f_1. This relationship continues for larger fragment sizes, with a general form of:

$$p_i = \frac{f_i}{1 - \sum_{j=0}^{i-1} f_j}$$

If this condition is considered for the largest fragment detected with length n base pairs, we then have:

$$p_n = \frac{f_n}{1 - \sum_{j=0}^{n-1} f_j}$$

However, because f_i form a probability distribution over the n fragment sizes, we also have $\sum_{j=0}^{n} f_j = 1$, and thus $1 - \sum_{j=0}^{n-1} f_j = f_n$, and so, regardless of the value of f_n, $p_n = 1$. This effect also serves to increase the normalised probability of breaks in the vicinity of the iodine. This is illustrated in the main text in Figure 6, where even when

beginning from SSB with a known probability distribution from physical data, normalising it in this fashion leads to a significantly different SSB distribution, in terms of both scale and shape in the vicinity of the iodine. This unfortunately prevents many of the estimates from being used for absolute calibration within this work.
